# Retraction: Alcohol drinking alters stress response to predator odor via BNST kappa opioid receptor signaling in male mice

**DOI:** 10.7554/eLife.74986

**Published:** 2021-11-02

**Authors:** Lara S Hwa, Sofia Neira, Meghan E Flanigan, Christina M Stanhope, Melanie M Pina, Dipanwita Pati, Olivia J Hon, Waylin Yu, Emily Kokush, Rachel Calloway, Kristen Boyt, Thomas L Kash

Hwa LS, Neira S, Flanigan ME, Stanhope CM, Pina MM, Pati D, Hon OJ, Yu W, Kokush E, Calloway R, Boyt K, Kash TL. 2020. Alcohol drinking alters stressresponse to predator odor via BNST kappa opioid receptor signaling in male mice. *eLife* **9**:59709. doi: 10.7554/eLife.59709

Published 21 July 2020

The authors are retracting this *eLife* paper based on error in methods and data reporting, which they identified following publication, that cast doubt on the conclusions.

Following publication several co-authors (SN, MP, OH, DP, MF) came to the senior author (TK) with concern about an error in the published data set. The initial error identified was the inclusion of an incorrect group for measurement of blood ethanol content (Figure 1D, female dynorphin reporter mice included instead of male wild-type mice). Following the identification of this initial error, the first author (LH), co-authors (SN, MP, OH, DP, MF, WY, CS) and senior (TK) deeply examined the results and identified several more errors. Notably, these errors occurred across three major domains:

1) Inclusion of female mice where only male mice were reported to be used (Figure 1D Blood Ethanol Concentration, Figure 2 Fos IHC, Figure 4 Ephys). Specifically:

Figure 1D: Blood Ethanol Concentrations. The current figure legend states, "Blood ethanol concentrations (mg/dl) correlated with EtOH intake (g/kg/2 hr) in a subset of mice." Figure 1 shows male C57BL/6J mice, but these blood ethanol concentrations were from female Pdyn-GFP mice from a different study.

Figure 2: One female Dyn-GFP mouse was included in this figure.

Figure 4: Two of the IA mice are female, Three of the Water/TMT mice are female.

2)Inclusion of mice with drinking outside the stated alcohol drinking range (actual: 6–8 weeks vs stated: 6 weeks) and withdrawal dates (actual: 2–82 days vs stated: 7–10 days): Specifically:

Figure 2: The Figure shows Pdyn-GFP mice drank alcohol for 6 weeks, but Pdyn-GFP mice that drank EtOH up to 8 weeks were included in the analysis. In addition, mice were included with withdrawal times ranging from 4 to 14 days.

Figure 4: The Figure shows Pdyn-GFP mice drank for 6 weeks, but as above experiment we identified that several mice that drank for up to 7 weeks. In addition, the withdrawal time points were broader (one mouse at 3 days, one mouse at 28 days, one mouse at 29 days, one mouse at 60 days) instead of the reported 7–10 days post-EtOH recording time.

Figure 6: The Figure shows Pdyn-GFP mice drank for 6 weeks, but as above, several mice that drank 7 weeks were included in the analysis. Additionally, the withdrawal time points were broader (one mouse at 21 days, one mouse at 82 days) instead of the reported 7–10 days post-EtOH recording time.

Figure 6 Supplemental 1: The withdrawal time points were outside the stated range (one mouse at 21 days, one mouse at 22 days, one mouse at 28 days)

3)Errors in the data presented in several of the behavioral figures. The data plotted in several of the published figures did not match the raw data values obtained directly from the ethovision tracking files (Figure 1G, Figure 3A, Figure 5I, Figure 7K). When the raw data obtained from ethovision was re-analyzed, there were multiple changes in statistical significance, leading to an overall change in the interpretation of the results. Specifically:

In Figure 1F,G,H, we identified that the norBNI treated groups were swapped, with the water norBNI mice being shown as IA norBNI mice, and the IA norBNI mice being shown as water norBNI mice. The statistical differences across groups were not different.

In Figure 1G: 2 values for TMT contact were different between the raw values provided by ethovision and the values plotted in prism. When the raw values were analyzed there was no longer a statistical difference between groups. In addition, in the published manuscript, the factors of drinking and TMT were swapped in the analysis.

Figure 3A: 7 values for TMT contact were different between the raw values provided by ethovision and the values plotted in prism. When the raw values were analyzed there was no longer a statistical main effect.

Figure 5I: 2 values for TMT contact were different between the raw values provided by ethovision and the values plotted in prism, noted that this was due to hand-scoring. With 1 of these differences the values do not match between excel and prism. When the values from excel are plotted and analyzed (including hand scored corrections) there is a main effect of drinking on TMT contact, but no other significant differences.

Figure 7K: 5 values for TMT contact were different between the raw values provided by ethovision and the values plotted in prism. 1 of these values is due to a mouse being analyzed twice. When the values from excel are plotted, there is are no longer any statistical differences between groups.

The changes that would be required to correct the paper are sufficiently extensive that its overall conclusions must currently be considered to be in doubt. All of the authors are in agreement that retraction is the appropriate course of action.

